# The Attentive Cursor Dataset

**DOI:** 10.3389/fnhum.2020.565664

**Published:** 2020-11-16

**Authors:** Luis A. Leiva, Ioannis Arapakis

**Affiliations:** ^1^Department of Communications and Networking, Aalto University, Espoo, Finland; ^2^Telefónica Research, Madrid, Spain

**Keywords:** aimed movements, attention, demographics, web search, mouse cursor

## 1. Introduction

We introduce a large-scale dataset of mouse cursor movements that can be used to predict user attention, infer demographics information, and analyze fine-grained movements. Attention is a finite resource, so people spend their time on things they find valuable, especially when browsing online. Objective measurements of attentional processes are increasingly sought after by researchers, advertisers, and other key stakeholders from both academia and industry. With every click, digital footprints are created and logged, providing a detailed record of a person's online activity. However, click data provide an incomplete picture of user interaction, as they inform mainly about a users' end choice. A user click is often preceded by several valuable interactions, such as scrolling, hovers, aimed movements, etc. and thus having access to this kind of data can lead to an overall better understanding of the user's cognitive processes. For example, previous work has evidenced that when the mouse cursor is motionless, the user is processing information (Hauger et al., 2011; Huang et al., 2011; Diriye et al., 2012; Boi et al., 2016), i.e., essentially “users first focus and then execute actions” (Martín-Albo et al., 2016). We have collected mouse cursor tracking logs from near 3K subjects performing a transactional search task that together account for roughly 2 h worth of interaction data. Our dataset has associated attention labels and five demographics attributes that may help researchers to conduct several analysis, like the ones we discuss later in this section.

Research in mouse cursor tracking has a long track record. Chen et al. (2001) were among the first ones to note a relationship between gaze position and cursor position during web browsing. Mueller and Lockerd (2001) investigated the use of mouse tracking to create compelling visualizations and model the users' interests. It has been argued that mouse movements can reveal subtle patterns like reading (Hauger et al., 2011) or hesitation (Martín-Albo et al., 2016), and can help the user regain context after an interruption (Leiva, 2011a). Others have also noted the utility of mouse cursor analysis as a low-cost and scalable proxy of eye tracking (Huang et al., 2012; Navalpakkam et al., 2013). Several works have investigated closely the utility of mouse cursor data in web search (Arapakis et al., 2015; Lagun and Agichtein, 2015; Liu et al., 2015; Arapakis and Leiva, 2016; Chen et al., 2017) and web page usability evaluation (Arroyo et al., 2006; Atterer et al., 2006; Leiva, 2011b), two of the most prominent use cases of this technology. Mouse biometrics is another active research area that has shown promise in controlled settings (Lu et al., 2017; Krátky and Chudá, 2018). Researchers have started to analyze mouse movements on websites for the detection of neurodegenerative disorders (White et al., 2018; Gajos et al., 2020). In practice, commercial web search engines often use mouse cursor tracking to improve search results (Huang et al., 2011, 2012), optimize page design (Leiva, 2012; Diaz et al., 2013), and offer better recommendations to their users (Speicher et al., 2013). In what follows, we provide a brief survey of what others have accomplished by analyzing mouse cursor movements in web search tasks. These analyses highlight potential use cases of our dataset, thereby allowing researchers to investigate similar environments and behaviors.

### 1.1. Inferring Interest

For a long time, commercial search engines have been interested in how users interact with Search Engine Result Pages (SERPs), to anticipate better placement and allocation of ads in sponsored search or to optimize the content layout. Early work considered simple, coarse-grained features derived from mouse cursor data to be surrogate measurements of user interest (Goecks and Shavlik, 2000; Claypool et al., 2001; Shapira et al., 2006). Follow-up research transitioned to more fine-grained mouse cursor features (Guo and Agichtein, 2008, 2010) that were shown to be more effective. These approaches have been directed at predicting open-ended tasks like search success (Guo et al., 2012) or search satisfaction (Liu et al., 2015). Mouse cursor position is mostly aligned to eye gaze, especially on SERPs (Guo and Agichtein, 2012; Lagun et al., 2014a), and that can be used as a good proxy for predicting good and bad abandonment (Diriye et al., 2012; Brückner et al., 2020).

### 1.2. Inferring Visual Attention

Mouse cursor tracking has been used to survey the visual focus of the user, thus revealing valuable information regarding the distribution of user attention over the various SERP components. Despite the technical challenges that may arise from this analysis, previous work has shown the utility of mouse movement patterns to measure within-content engagement (Arapakis et al., 2014a; Carlton et al., 2019) and predict reading experiences (Hauger et al., 2011; Arapakis et al., 2014b). Lagun et al. (2014a) introduced the concept of motifs, or frequent cursor subsequences, in the estimation of search result relevance. Similarly, Liu et al. (2015) applied the motifs concept to SERPs and predicted search result utility, searcher effort, and satisfaction at the search task level. Boi et al. (2016) proposed a method for predicting whether the user is actually looking at the content pointed by the cursor, exploiting the mouse cursor data and a segmentation of the web page contents. Lastly, Arapakis and Leiva (2016) investigated user engagement with direct displays on SERPs and provided further evidence that supports the utility of mouse cursor data for measuring user attention at a display-level granularity (Arapakis and Leiva, 2020; Arapakis et al., 2020).

### 1.3. Inferring Emotion

The connection between mouse cursor movements and the underlying psychological states has been a topic of research since the early 90s (Card et al., 1987; Accot and Zhai, 1997). Some studies have investigated the utility of mouse cursor data for predicting the user's emotional state. For example, Zimmermann et al. (2003) investigated the effect of induced affective states on the motor-behavior of online shoppers and found that the total duration of mouse cursor movements and the number of velocity changes were associated to the experienced arousal. Kaklauskas et al. (2009) created a system that extracts physiological and motor-control parameters from mouse cursor interactions and then triangulated those with psychological data taken from self-reports, to correlate the users' emotional state and productivity. In a similar line, Azcarraga and Suarez (2012) combined electroencephalography signals and mouse cursor interactions to predict self-reported emotions like frustration, interest, confidence and excitement. Yamauchi (2013) studied the relationship between mouse cursor trajectories and generalized anxiety in human subjects. Lastly, Kapoor et al. (2007) predicted whether a user experiences frustration, using an array of affective-aware sensors.

### 1.4. Inferring Demographics

Prior work has linked age with motor control and pointing performance in tasks that involve the use of a computer mouse (Walker et al., 1997; Bohan and Chaparro, 1998; Hsu et al., 1999; Smith et al., 1999; Jastrzembski et al., 2003; Lindberg et al., 2006). Overall, aging is marked by a decline in motor control abilities, therefore it is expected to affect the users' pointing performance and, by extension, how they move the computer mouse. For example, Smith et al. (1999) observed that older people incurred in longer mouse movement times, more sub-movements, and more pointing errors than the young. These findings underline potential age effects on the way a mouse device is used in an online search task. Prior research has also noted sensory-motor differences due to gender (Landauer, 1981; Chen and Chen, 2008; Yamauchi et al., 2015), such as significant variation in the cursor movement distance, pointing time, and cursor patterns. The cause of these variations has been attributed to gender-based differences in how users move a mouse cursor or to different cognitive mechanisms (perceptual and spatial processes) involved in motor control.

Others have also examined the extent to which mouse cursor movements can help identify gender and age (Yamauchi and Bowman, 2014; Kratky and Chuda, 2016; Pentel, 2017), however the experimental settings have limited generalizability, either because the tasks are not well-connected to typical activities that users perform online, such as web search, because the data include multiple samples per participant, thereby increasing the risks of information leakage, or because researchers could not verify their ground-truth data. In our dataset, we limit the training samples to exactly one mouse cursor trajectory per participant, who are verified, high-quality crowdworkers.

## 2. Method

We ran an online crowdsourcing study that reproduced the conditions of a transactional search task. Participants were presented with a simulated information need that explained that they were interested in purchasing some product for them or a friend. Overall, the study consisted of three parts, to be described later: (1) pre-task guidelines, (2) the web search task, and (3) a post-task questionnaire.

### 2.1. Participants

We recruited participants from the Figure Eight crowdsourcing platform^1^. They were of mixed nationality (e.g., American, Belgian, British, German) and had diverse educational backgrounds (see Table 1). All participants were proficient in English and were experienced (Level 3) contributors, i.e., they had a proven track record of successfully completed tasks and of a different variety, thus being considered very reliable contributors.

**Table 1:** Demographics information from our dataset.

**Age**	**Count**	**Gender**	**Count**	**Nationality**	**Count**	**Education**	**Count**	**Income**	**Count**
18–23	380	Male	1,605	USA	1,755	High school	593	<25K	881
24–29	716	Female	1,118	VEN	251	College	472	25–34K	446
30–35	590	NA	14	GBR	209	Bachelor's	704	35–49K	367
36–41	417			CAN	66	Graduate	499	50–74K	394
42–47	223			EGY	37	Master's	399	75–99K	249
48–53	174			UKR	31	Doctorate	30	100–149K	145
54–59	132			IND	29	NA	40	150–249K	42
60–65	63			SRB	27			>250K	23
+66	24			RUS	25			NA	190
NA	18			…					

### 2.2. Materials

Starting from Google Trends^2^, we selected a subset of the Top Categories and Shopping Categories that were suitable representatives of transactional tasks. Then, we extracted the top search queries issued in the US during the last 12 months. Next, we narrowed down our search query collection to 150 representative popular queries. The final collection of transactional queries was repeated as many times needed to produce the desired number of search sessions for the final dataset.

Using this final selection of search queries, we produced the static version of the corresponding Google SERPs and injected custom JavaScript code that allowed us to capture all client-side user interactions. For this, we used EvTrack^3^, an open source JavaScript event tracking library derived from the smt2ϵ mouse tracking system (Leiva and Vivó, 2013). EvTrack can capture browser events either via event listeners (the event is captured as soon as it is fired) or via event polling (the event is captured at fixed-time intervals). We captured mousemove events via event polling, every 150 ms to avoid unnecessary data overhead (Leiva and Huang, 2015), and all the other browser events (e.g., load, click, scroll) via event listeners. Whenever an event was recorded, we logged the following information: mouse cursor position (*x* and *y* coordinates), timestamp, event name, XPath of the DOM element that relates to the event, and the DOM element attributes (if any).

All queries triggered some form of advertisements on the SERPs, according to three different formats: “native” (organic ads) or “bundled” (direct display ads). All SERPs included one or more native ads together with one bundled ad. The native advertisements could appear either at the top or bottom position of the SERP, whereas the bundled ads could appear either at the top-left or top-right position. We ensured that only one ad was visible per condition and participant at a time. This was possible by instrumenting each downloaded SERP with custom JavaScript code that removed all ads excepting one that would be selected for a given participant. In any case, native bottom-most ads were not shown to the participants.

### 2.3. Pre-task Guidelines

Participants were instructed to read carefully the terms and conditions of the study which, among other things, informed them that they should perform the task from a desktop or laptop computer using a computer mouse (and refrain from using a touchpad, tablet, or mobile device) and that their browsing activity would be logged. Moreover, participants consented to share their browsing data and their (anonymized) responses for later analysis.

Participants were asked to act naturally and choose anything that would best answer a given search query, since all “clickable” elements (e.g., result links, images, etc.) on the SERP were considered valid answers. The instructions were followed by a brief search task description using this template: “*You want to buy* <*noun*> *(for you or someone else as a gift) and you have submitted the search query* <*noun*> *to Google Search. Please browse the search results page and click on the element that you would normally select under this scenario*.” The template was populated with the corresponding <noun> entities, based on the assigned query.

Participants were allowed as much time as they needed to examine the SERP and proceed with the search task, which would conclude whenever they clicked on any SERP element. The payment for the participation was $0.20. Participants could also opt out at any moment, in which case they were not compensated. Each participant could take the study only once.

### 2.4. Task Procedure

Each participant was presented with a search task description, then provided with a predefined search query (selected at random from our pool of queries) and the corresponding SERP, and they were asked to click on any element of the page that best solved the task. This way, we ensured that participants interacted with the same pool of web search queries and avoided any unaccounted systematic bias due to query quality variation. All possible combinations of query and ad style (i.e., format and position) were pre-computed so that whenever a new user accessed the study, they were assigned one of these combinations at random.

Participants accessed the instrumented SERPs through a dedicated web server that did not alter the look and feel of the original SERPs. This allowed us to capture fine-grained user interactions while ensuring that the content of the SERPs remained consistent with the original version. Each participant was allowed to perform the search task only once to avoid introducing possible carry over effects and, thus, altering their browsing behavior in subsequent search tasks. In sum, each participant was exposed only to a single condition; i.e., a unique combination of query and ad style. Finally, at the end of the study participants had to copy a unique code and paste it on Figure Eight in order to have their job validated.

### 2.5. Post-task Questionnaire

Upon concluding the search task, participants were asked to answer a series of questions. The questions were forced-choice type and allowed multi-point response options.

The first question asked the degree to which the user noticed the advertisements shown on the SERP: *While performing the search task, to what extent did you pay attention to the advertisement?* We used a 5-point Likert-type scale to collect the labels: 1 (“Not at all”), 2 (“Not much”), 3 (“I can't decide”), 4 (“Somewhat”), and 5 (“Very much”). In practice, these scores should be collapsed to binary labels (true/false), but we felt it was necessary to use a 5-point Likert-type scale for several reasons. First, using 2-point scales often results in highly skewed data (Johnson et al., 1982). Second, it is important to leave room for neutral responses, because some users may not want to say one way or another, otherwise this can produce response biases. But 3-point scales can lead more users to stay neutral, because the remaining options can be seen as “too extreme.” Therefore, we opted for a 5-point scale, which leaves more room for “soft responses” and in addition is easy to understand. With this scoring scheme, therefore, we are confident that eventual binary labels would actually reflect positive and negative user votes.

The questionnaire also comprised the following demographics-related questions:

1. *What is your gender?* [Male, Female, Prefer not to say]
2. *What is your age group?* [18–23, 24–29,…, 60–65, +66, Prefer not to say]
3. *What is your native language?* [Pull-down list, Prefer not to say]
4. *What is your education level?* [High school, College,…, Doctorate, Prefer not to say]
5. *What is your current income?* [25K, 35K,…, 250K, Prefer not to say]

## 3. Validation and Filtering

Crowdsourcing studies offer several advantages over *in-situ* methods of experimentation (Mason and Suri, 2012), such as access at a larger and more diverse pool of participants with stable availability, collection of real usage data at a relatively large scale, and a low-cost alternative to the more expensive laboratory-based experiments. On the downside, experimenters have to account for potential threats to ecological validity, distractions in the physical environment of the participant, and privacy issues, to name a few. Still, crowdsourcing allows for exploring a wider range of parameters in a more controlled manner as compared to in-the-wild large-scale studies.

We collected self-reported ground-truth labels in a similar vein to previous work (Feild et al., 2010; Lagun et al., 2014b; Liu et al., 2015; Arapakis and Leiva, 2016) which also administered post-task questionnaires. To mitigate and discount low-quality responses, several preventive measures were put into practice, such as introducing test (gold-standard) questions to our tasks, selecting experienced contributors with high accuracy rates, and monitoring their task completion time, thus ensuring the internal validity of our experiment.

Starting from a set of 3,223 participants who initially accessed the study, we filtered automatically those who did not finish it (138 cases) as well as participants who did not move their mouse at all (176 cases). We concluded to a dataset with 2,909 observations comprising at least one mouse movement, together with their associated browser's and user's metadata. See Table 1 for a summary of the available demographics information.

There are 92 unique combinations of query and ad style, each of which assessed by 32 users on average (*SD* = 17 users). There are 1,942 observations from the attended condition (self-reported Likert-type score ≥4), 776 observations from the non-attended condition (score ≤ 2), and 191 observations from the neutral condition (score of 3). The average mouse cursor trajectory has 15.78 coordinates (*SD* = 16.5, min = 1, max = 222), which is around the same order of magnitude as reported in similar studies (Huang et al., 2011; Leiva and Huang, 2015; Arapakis and Leiva, 2016).

Excepting the automatic filtering procedure explained above, our data is in raw form and therefore some columns require further processing. For example, most columns pertaining demographics information are stored as integers, therefore researchers should consult Table 1 to retrieve the corresponding categorical labels. We also recommend researchers to apply other filtering methods, depending on the nature of their experiments, such as collapsing the ground-truth attention labels from the original 1–5 scale to a binary scale (Arapakis and Leiva, 2020; Arapakis et al., 2020) or ignoring cursor trajectories having <5 coordinates, which in most cases would correspond to 1 s of interaction data.

### 3.1. Data Format

The Attentive Cursor dataset includes the following resources:

1. A folder with mouse tracking log files, as recorded by the EvTrack software:
  - Browser events: space-delimited files (CSV) with information about each event type (8 columns).
  - Browser metadata: XML files with information about the user's browser (e.g., viewport size).
2. A TSV file with ground-truth labels (4 columns).
3. A tab-delimited file (TSV) with user's demographics and stimulus condition (12 columns).
4. A folder with all SERPs in HTML format.
5. A README file with a detailed explanation of each resource.

**Figure 1:**
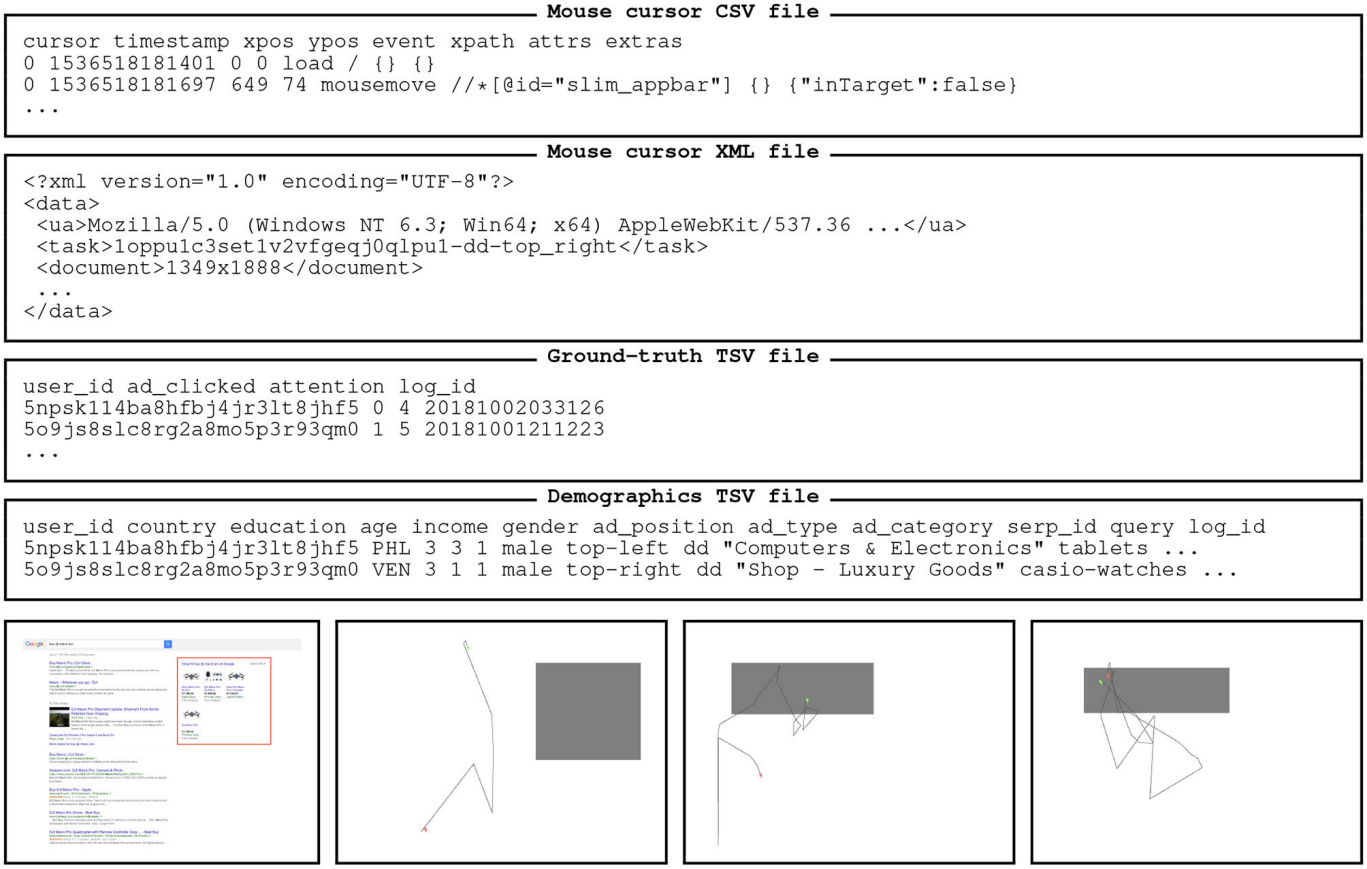
File content samples (top) and SERP snapshots with mouse cursor trajectories (bottom). An ellipsis (…) denotes an intentional omission of some data, for brevity's sake. The gray-colored rectangles in the bottommost figures denote the different ad types, from left to right: right-aligned bundled ad, left-aligned bundled ad, and native ad.

Figure 1 provides some examples of the kind of data that researchers can find in our dataset. We provide the URL to the repository in the “Data Availability Statement” section below.

## 4. Conclusion

We have presented a large-scale, in-the-wild dataset of mouse cursor movements in web search, with associated ground-truth labels about user's attention and demographics attributes. The dataset represents real-world behavior of individuals completing a transactional web search task. What makes this dataset both unique and challenging is the fact that there is only one observation per user. It is not possible to leak information from any data splits; e.g., training, validation, and testing splits typically used in machine learning studies. It is our hope that the dataset will foster research in several scientific domains, Including, e.g., information retrieval, movement science, and psychology.

## Data Availability Statement

The raw data supporting the conclusions of this article will be made available by the authors, without undue reservation.

## Ethics Statement

Ethical review and approval was not required for the study on human participants in accordance with the local legislation and institutional requirements. The patients/participants provided their written informed consent to participate in this study.

## Author Contributions

All authors listed have made a substantial, direct and intellectual contribution to the work, and approved it for publication.

## Conflict of Interest

IA was employed by the company Telefonica Research, though no payment or services from the institution has been received or requested for any aspect of the submitted work. The remaining author declares that the research was conducted in the absence of any commercial or financial relationships that could be construed as a potential conflict of interest.

## Footnotes

^1^https://www.figure-eight.com

^2^https://trends.google.com/trends/

^3^https://github.com/luileito/evtrack
